# Effect of dietary fiber on trimethylamine-N-oxide production after beef consumption and on gut microbiota: MEATMARK – a randomized cross-over study

**DOI:** 10.1038/s41430-025-01636-8

**Published:** 2025-06-19

**Authors:** Melanie Haas, Beate Brandl, Klaus Neuhaus, Susanne Wudy, Karin Kleigrewe, Hans Hauner, Thomas Skurk

**Affiliations:** 1 Core Facility Human Studies, ZIEL Institute for Food & Health, Technical University of Munich, Freising, Germany; 2 Core Facility Microbiome, ZIEL Institute for Food & Health, Technical University of Munich, Freising, Germany; 3 Bavarian Center for Biomolecular Mass Spectrometry (BayBioMS), TUM School of Life Sciences, Technical University of Munich, Freising, Germany; 4 School of Medicine and Health, Technical University of Munich, Munich, Germany; 5 Else Kröner-Fresenius-Center of Nutritional Medicine, TUM School of Life Sciences, Technical University of Munich, Freising, Germany

**Keywords:** Risk factors, Biomarkers

## Abstract

**Background/Objectives:**

The gut-microbiota-dependent metabolite trimethylamine-N-oxide (TMAO) has been linked to cardiovascular disease (CVD) risk, while dietary fiber is associated with reduced CVD risk and improved gut health. Considering these associations, we conducted a randomized, double-blind, pilot study to investigate the influence of fiber supplementation on intestinal TMAO formation after beef consumption.

**Subjects/Methods:**

13 volunteers underwent a two-week dietary fiber and placebo intervention. We assessed the effect of fiber supplementation on the gut microbiota and gene abundance of the enzyme cutC, a key enzyme for microbial TMA formation, a precursor for TMAO. We measured the TMAO response following beef consumption after the two-week intervention. We also examined the role of hepatic enzyme FMO3 on TMAO plasma levels.

**Results:**

Although overall TMAO production did not change between the dietary fiber and placebo group (*p*-value = 0.26, 95% CI), subgroup analysis revealed that fiber supplementation attenuated TMAO formation following beef intake in participants with lower habitual meat consumption ( <3 times/week, *p*-value = 0.029, 95% CI). Furthermore, fiber intervention significantly downregulated microbial cutC gene abundance (*p* = 0.034, 95% CI), suggesting a mechanism by which fiber might reduce plasma TMAO levels. While dietary fiber intervention did not alter TMAO production across all participants, it showed a potential effect in individuals with lower habitual meat intake. The observed downregulation of cutC gene abundance suggests a mechanism for the beneficial impact of fiber on TMAO formation.

**Conclusion:**

These findings support the role of a high-fiber, low-meat diet as a promising strategy to mitigate TMAO-related CVD risk.

**Figure:**
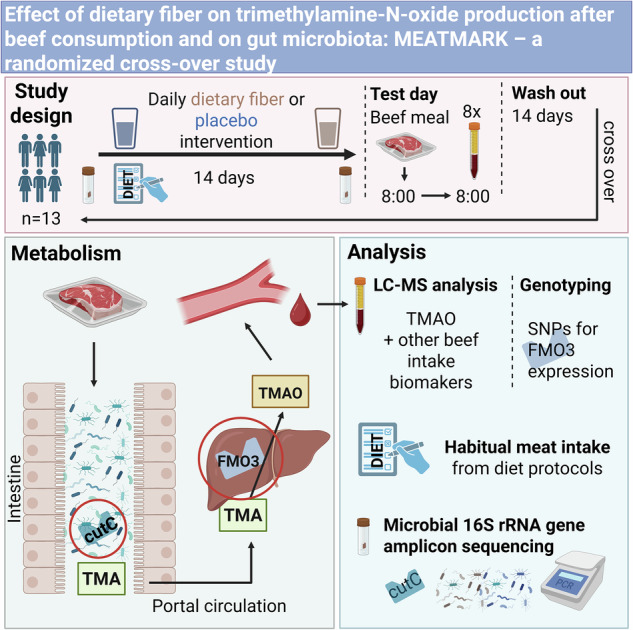
Graphical abstract of the MEATMARK study. Created with BioRender.com. Haas, M. (2025) https://BioRender.com/x12v771.

## Introduction

TMAO, a gut microbiome-dependent metabolite, has recently emerged as a potential cardiovascular risk factor [1–4]. Meta-analyses and epidemiological studies suggest an association between elevated TMAO levels and an increased incidence of cardiovascular events, including atherosclerosis, thrombosis, and atrial fibrillation [3–8]. However, the link between TMAO and CVD remains inconsistent [9, 10] and the exact mechanisms by which TMAO may increase this risk are not yet fully understood [11].

TMAO production is influenced by various endogenous and exogenous factors, including dietary patterns [12, 13], age [14], gut microbiome composition [12] and the expression of specific microbial and hepatic enzymes [15, 16]. Yet, the relative contribution of these factors to TMAO formation remains unclear, highlighting the need for further research into strategies for reducing TMAO levels and, through this, potentially mitigating cardiovascular risk [17].

Physiologically, TMAO functions as an osmolyte, playing a crucial role in cellular homeostasis by stabilizing protein folds, nucleic acids, and maintaining cell volume [18, 19]. It also contributes to lipid metabolism, influencing cholesterol transport and distribution [19]. Nonetheless, its association with chronic diseases is complex. Elevated TMAO levels have been linked to systemic inflammation, platelet hyperreactivity, atherosclerosis progression, renal fibrosis, and altered bile acid metabolism, all of which contribute to CVD, renal disease, and diabetes [2, 17, 19]. While the precise mechanisms remain incompletely understood, evidence suggests that TMAO may modulate cholesterol and sterol metabolism, suppress reverse cholesterol transport, and influence angiotensin II signaling, a key regulator of circulatory homeostasis [11, 12, 19, 20].

TMAO can be directly obtained from dietary sources such as fish and seafood, but the majority of circulating TMAO originates from a meta-organismal pathway involving dietary precursors like choline, L-carnitine, and betaine [1, 2, 21]. These precursors, primarily found in red meat, eggs, and dairy products, are metabolized by gut microbiota into trimethylamine (TMA) [12, 17, 22]. Two key enzymatic pathways facilitate microbial TMA production. The choline TMA-lyase enzyme (*cutC*), which metabolizes choline and the Rieske-type oxygenase/reductase enzyme system (*cntA*), which processes L-carnitine [23]. TMA is subsequently absorbed and transported to the liver, where it is oxidized by flavin-containing monooxygenases (*FMOs*), predominantly *FMO3*, to form TMAO [24].

Dietary patterns strongly seems to influence TMAO metabolism, as seen in studies comparing vegans and omnivores, where lower habitual meat intake is associated with reduced TMAO production [12]. Given the growing evidence linking diet, microbiota composition, and TMAO metabolism, novel dietary strategies are being explored to modulate microbiome activity in a beneficial way [25].

One promising approach is dietary fiber supplementation, which has been shown to exert beneficial prebiotic effects by modifying gut microbiota composition [26, 27]. Additionally, dietary fiber contributes to cardiovascular health, with extensive evidence supporting its role in reducing CVD risk [28–30].

Beyond dietary factors, host genetics may also influence TMAO metabolism. Among hepatic *FMOs*, *FMO3* is the primary enzyme responsible for converting TMA into TMAO. Mutations in the *FMO3* gene have been linked to trimethylaminuria (“fish odor syndrome”), a rare genetic disorder characterized by impaired TMA oxidation, leading to TMA accumulation and a distinctive fish-like body odor [16, 24].

Interestingly, while no direct link between trimethylaminuria and CVD has been established [16], this raises the question of whether genetic variations in the *FMO3* gene, particularly those influencing *FMO3* expression, could contribute to elevated TMAO levels and, consequently, an increased risk of CVD. Evidence suggests that higher *FMO3* expression correlates with increased plasma TMAO concentrations [31] and mechanistic studies in mice indicate that *FMO3* suppression reduces TMAO levels and atherosclerosis progression [32]. Although research in this area remains limited in humans, some studies have investigated potential *FMO3* variants. For example, the missense variant *rs2266780* has been linked to an increased risk of CVD [33, 34]. Özçelik et al. reported that heterozygous carriers of this variant have a higher risk of hypertension-related ischemic stroke, emphasizing the need for further genetic research [33].

This pilot study aimed to investigate the effects of dietary fiber supplementation on gut microbiota composition, microbial *cutC* gene abundance, and TMAO production following beef consumption. We examined whether fiber supplementation could modulate microbial TMA formation and subsequently influence plasma TMAO levels. Additionally, we assessed the role of hepatic *FMO3* enzyme activity in TMAO metabolism and considered meat intake patterns as potential influencing factors. This exploratory approach provides comprehensive insights into the role of diet in modulating TMAO levels as a potential strategy for reducing CVD risk.

## Methods

Procedures for the intervention study were followed in accordance with the ethical standards of the Helsinki Declaration and were reviewed and approved by the ethics committee of the School of Medicine at the Technical University of Munich, Germany (331/20S). All study participants had provided written informed consent. The study was registered in the German Clinical Trials Register (DRKS): DRKS00021291.

### Study design

The MEAT intake bioMARKer (MEATMARK) study is a six-week randomized, cross-over, double-blind pilot study involving thirteen healthy volunteers (6 females, 7 males). As there is currently no published data specifically examining the relationship between dietary fiber intervention and TMAO production, we designed an exploratory pilot study to investigate this potential link. The aim was to generate preliminary data that could serve as a basis for larger, more powered studies in the future. To determine the appropriate number of subjects, we followed the guidelines for human experimentation established in the work of Ulaszewska et al. [35]. Each participant underwent two intervention phases, each lasting two weeks, during which they consumed either the dietary fiber supplement or a placebo made of maltodextrin (see “Study Product” for details on the products used). The order of the intervention was randomized; the randomization was performed with Excel 2016. A random order of our two sets (intervention-placebo, placebo-intervention) was generated for each participant using the function ( = INDEX(); RANDBETWEEN(1,2)).

**Fig. 1:**
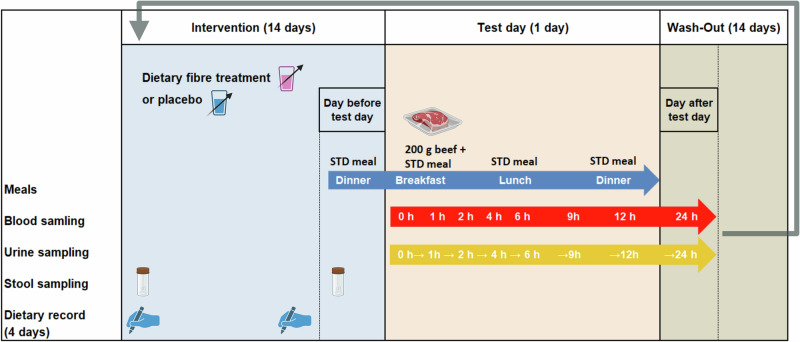
Study design of the MEATMARK study. One study cycle is shown; each participant underwent two cycles distinguished by the intervention (either dietary fiber supplement or placebo). The entire cross-over study period lasted six weeks, consisting of two two-week interventions, followed by a study day and separated by a two-week wash-out period. The STD meal was also provided the evening before test day and for lunch and dinner on test day (blue arrow). Blood (red arrow), urine (yellow arrow), and stool were sampled at defined time points and dietary records were taken on four consecutive days before and at the end of the intervention phase. Created with BioRender.com. Haas, M. (2025) https://BioRender.com/i99e952.

Following each intervention phase, participants arrived fasted at the study center in the morning of the test day and consumed a test meal composed of 200 g of beef, 125 g of rice, 30 g of margarine, and 1.5 g of salt to assess any differences in TMAO metabolism after fiber supplementation compared to placebo. A two-week washout period separated the two intervention phases, as illustrated in Fig. 1.

During the intervention phases, the fiber supplements, provided as powder, were suspended in water immediately prior to consumption. To acclimate to the high-fiber supplement, participants continuously increased their intake from 9 to 27 g of fiber or placebo over time. On the evening before a test day, participants received a standardized meal (STD meal) comprising 125 g of rice, 30 g of margarine, and 1.5 g of salt. For breakfast on intervention days, participants consumed 200 g *sous-vide* cooked beef alongside the STD meal. The STD was also provided for lunch and dinner on the intervention day.

On the test day, blood and urine samples were collected at defined intervals over a 24 h period, while stool samples were collected before and after each two-week intervention phase. Participants were instructed to document their food intake for four consecutive days before the intervention phase and for the last four days during the intervention phase and to categorize the stool according to the Bristol stool scale. Additionally, participants refrained from consuming foods containing TMAO or its precursors (including meat, fish, eggs, and foods containing L-carnitine) for two days prior to the intervention day.

### Study participants

**Fig. 2:**
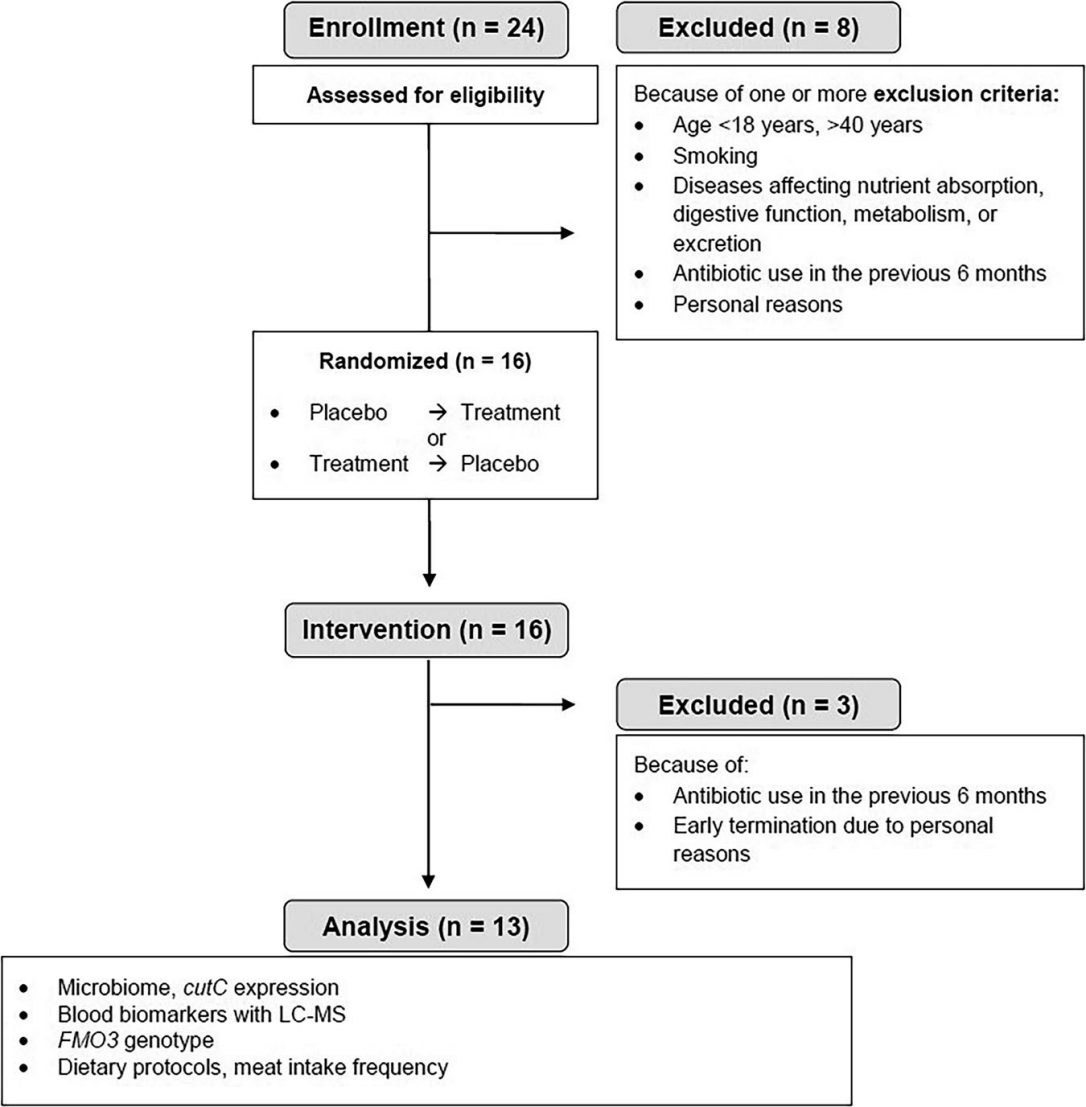
Consort flow diagram of study participants.

For this trial, we aimed to find healthy volunteers (18–40 years) via flyers at the Technical University of Munich, campus Freising. Screening started on August 10th, 2020, and continued until December 1st, 2020. The participants’ eligibility was assessed with a detailed screening questionnaire. Exclusion criteria were age <18 years and >40 years, smoking, diseases affecting nutrient absorption, digestive, metabolic, or excretory function, chronic diseases from the medical history (e.g. hepatitis B and C, diabetes mellitus), antibiotic use in the previous six months, regular medication intake (except oral contraceptives), pregnant and breastfeeding women, known allergies or food intolerances to any of the test food ingredients, diverticulitis and/or constipation in the medical history. Figure 2 depicts the participant flow chart of the MEATMARK study.

### Study products

The dietary fiber supplement, as well as the placebo product, were provided by J. Rettenmaier & Söhne GmbH + Co KG, (Rosenberg, Germany). Both products were supplied in powdered form and should be suspended in water for consumption. The type of this fiber was chosen as it is frequently used in foods and supplements [36, 37].

The dietary fiber product comprised 48% total dietary fiber, consisting of 87% wheat fiber, 7% psyllium, and 6% guar gum. The remaining components of the product included 42% isomaltulose, citric acid, sucralose, and some flavors and food colorants. Conversely, the placebo product contained a total dietary fiber content of 10%, supplemented by 25% maltodextrin, 7.5% vegetable potato flakes, 50% isomaltulose. The detailed composition is provided in the supplementary material (see Table S1**:** *Composition of study products* in the supplementary material).

Each product was packed in sachets of 19.05 g, corresponding to 9 g of total dietary fiber. Participants were instructed to increase their intake of dietary fiber progressively: from 9 g (days 1-2), to 18 g (days 3–6), and finally to 27 g (days 7–14). The recommended method of consumption was to dissolve the powder in 200 mL of water and to consume it throughout the day alongside a meal.

### Blood, buffy coat and fecal sampling

#### Plasma and buffy coat sampling

Blood samples were collected in the fasting state and 1, 2, 4, 6, 9, 12 and 24 h after the beef meal as breakfast on the test day. Plasma (EDTA K2 monovettes, Sarstedt, Nümbrecht, Germany) was collected and centrifuged at 1800 × *g* for 10 min at 4 °C. Serum was collected, allowed to clot for 30 min, and then centrifuged (2500 × *g* for 10 min at 4 °C). Buffy coat was sampled from EDTA tubes (EDTA K3 monovettes, Sarstedt) after it was centrifuged at 1800 × *g* for 10 min at room temperature. Plasma, buffy coat and serum were aliquoted and stored at −80 °C for later analysis.

#### Stool sampling

Before and at the end of every intervention phase (Fig. 1), the participants collected stool samples in two separate tubes according to a standardized procedure. One tube contained 8 mL DNA stabilization buffer (Stratec Molecular GmbH, Berlin, Germany). Participants were instructed to bring the samples to the study center within 6 h, where they were subsequently stored at −80 °C until analysis [37].

### Targeted amino acid and acylcarnitine measurements

Targeted amino acid and acylcarnitine measurements were performed using a QTRAP 5500 triple quadrupole mass spectrometer (Sciex, Darmstadt, Germany) coupled to an ExionLC AD (Sciex, Darmstadt, Germany) ultrahigh performance liquid chromatography system. A multiple reaction monitoring (MRM) method was performed based on Wudy et al. [38], with extension by the following analytes (see Table S2**:***MRM Transitions for Quantification of Amino Acids and Acylcarnitines* in the supplementary material). Briefly, for chromatographic separation of amino acids and acylcarnitines, a 2.1 × 100 mm, 100 Å, 1.7 μm, UPLC BEH amide column (Waters, Eschborn, Germany) was used with 5 mM ammonium acetate in water (eluent A) and 5 mM ammonium acetate in acetonitrile/water (95/5, v/v) (eluent B) as elution solvents both adjusted to pH 3 using acetic acid. An injection volume of 1 µL and a flow rate of 0.4 mL/min was used. The gradient elution started at 100% B and was held for 1.5 min. Afterward, the concentration was decreased to 92% B at 3.5 min and further reduced to 90% B at 7 min. At 10 min, 78% B was used and decreased to 65% B, further decreased to 2% B at 12 min, and held for 1 min. At 15.5 min, the column was equilibrated at starting conditions. The column oven was set to 40 °C, and the autosampler to 15 °C. Ions of amino acids and acylcarnitines were analyzed in the positive electrospray ionization mode. The electrospray voltage was set to 5500 V, curtain gas to 35 psi, ion source gas 1 to 55 psi, ion source gas 2 to 65 psi, and the temperature to 400 °C. The MRM parameters (Table S2) were optimized using commercially available standards. Data acquisition and instrumental control were performed with Analyst v1.7 software (Sciex, Darmstadt, Germany). The data were analyzed with MultiQuant v3.0.3 software (Sciex, Darmstadt, Germany).

### High-throughput 16S rRNA gene sequencing and *cutC* gene abundance from microbiome DNA

#### Microbial 16S rRNA gene amplicon sequencing

The 16S rRNA gene sequencing was conducted as described in Reitmeier et al. [39]. Briefly, DNA was isolated after bead-beating using guanidinium thiocyanate as denaturing agent and N-lauroylsarcosine for separation. Finally, polyvinylpyrrolidone was used to remove phenolic inhibitors from the DNA [40]. After RNase A digest, 12 ng of the isolated and cleaned DNA was used for library preparation in a 2-step PCR. The first PCR uses primer for the V3-V4 region [41]; 341 F, CCTACGGGNGGCWGCAG; 785r-ovh, GACTACHVGGGTATCTAATCC) with overhangs for the second PCR. The subsequent PCR adds barcodes and adapter for Illumina sequencing. PCR products were cleaned and sequenced in equimolar batches on a MiSeq using PE300 cartridges.

Raw reads were processed using the UPARSE approach implemented in IMNGS using SOTUs as output [42]. Spurious taxa were filtered at <0.25% abundance per sample [43]. Denoised amplicons were assigned to taxa using Silva v138. Reads were normalized to the lowest read number of a given sample as described. Alpha and beta-diversity, taxonomic binning, as well as correlations were assessed using the pipeline Rhea, a collection of R-scripts for 16S rRNA data analysis [44].

#### *CutC* gene abundance

The gene abundance of the *cutC* was measured by quantitative PCR (qPCR) using microbial DNA as a template. The primers used for *cutC* gene abundance were cutC-F (TTY GCI GGI TAY CAR CCN TT) and cutC-R (TGN GGY TCI ACR CAI CCC AT), previously described by Rath et al. [22]. As reference, we used the 16S rRNA gene, amplified with primers 16S-F (CCT ACG GGN GGC WGC AG) and 16S-R (GAC TAC HVG GGT ATC TAA TCC) according to Klindworth et al. [41]. All primers were synthesized and purified by Merck (Taufkirchen, Germany). Amplification was performed with 10 ng of template DNA using a LightCycler 480 (Roche Diagnostics, Mannheim, Germany) and the Maxima SYBR Green/ROX qPCR Master Mix (ThermoFisher Scientific, Waltham, Massachusetts, USA) according to the manufacturer. qPCR was conducted as follows to obtain the quantification cycles (Cq): an initial 95 °C step for 10 min was followed by 40 cycles of denaturation at 95 °C for 30 s; annealing at 52 °C for 45 s; and an extension step at 72 °C for 45 s. Melting curves were subsequently generated using the following program: 95 °C for 10 s, followed by 60 °C for 1 s, and a final continuous reading step of two acquisitions per second between 60 and 95 °C. Primer efficiency was found to be 1.87 and 1.74 for *cutC* and 16S rRNA, respectively. ΔΔCq values were calculated for *cutC.* Results were normalized to the 16S rRNA gene copies. For *cutC* gene abundance analysis, we performed a ΔΔCq calculation using the 16S rRNA gene as a reference. We then calculated the fold-change (FC) of *cutC* between conditions and compared post-intervention FC to the respective baseline controls.

### DNA Isolation and *FMO3* Genotyping

Double-stranded DNA was isolated from buffy coat using the DNeasy Blood & Tissue Kit with DNeasy Mini spin-columns (QIAGEN, Hilden, Germany) following the manufacturer’s protocol. For genotyping, three different SNPs of the *FMO3* gene (*rs2266780, rs909530*, and *rs909531*) have been examined for mutations by using a melting curve analysis with LightSNiP assays from TIB MOLBIOL (TIB MOLBIOL, Berlin, Germany) in a LightCycler 480 (Roche Diagnostics, Mannheim, Germany). We followed the manufacturer’s protocol except for the polymerase, where we used MyTaq DNA Polymerase (Bioline, Heidelberg, Germany). The SNPs were selected after a comprehensive literature review and a search in biomedical databases (gnomAD browser v2.1.1: https://gnomad.broadinstitute.org/ and GTEx portal: https://www.gtexportal.org). We selected the SNPs based on their effect on *FMO3* expression and their abundance in population.

### Dietary protocols and subgroup analysis

The study participants were instructed to record their food consumption before the intervention phase and at the end of the intervention phase on four consecutive days. The energy content and macronutrient composition of the diets were calculated using the OptiDiet Plus software (Version 5.1.2.046, GOE GmbH, Linden, Germany).

Subgroups were stratified according to meat intake for additional analysis of plasma TMAO. Therefore, participants were asked how often meat and meat products were consumed on a regular basis. Less than three times a week was classified as ‘occasional’ (meat intake) and eating meat more than three times a week was classified as ‘regular’ (meat intake).

### enable study

To address our research question further, we performed *FMO3* genotyping (see “DNA isolation and *FMO3* genotyping”) along with LC-MS analysis (plasma TMAO) and results from usual intake and food frequency questionnaires (FFQ). For this purpose, we took advantage of our well-phenotyped cross-sectional *enable* study, which can be found elsewhere [36, 45].

Briefly, the phenotyping program of different *enable* age cohorts included, among others, anthropometry, body composition analysis, health and functional status, and assessment of dietary intake including food preferences and aversions. 459 healthy volunteers from different age groups including young adults (18–25 years; *n* = 94), middle-aged adults (40–65 years “middle-agers,” *n* = 205), and older adults (75–85 years; *n* = 160) underwent this program.

Additionally, we measured *cutC* gene abundance in stool samples from a nested intervention study with middle-agers presenting an elevated waist circumference ( > 102 cm in males, >88cm in females), indicating a higher cardiometabolic risk, was used to (see “High-Throughput 16S rRNA Gene Sequencing and *cutC* Gene Abundance from Microbiome DNA”). Samples and study design from this “Freising Fiber Acceptance Study” were recently described by Brandl et al. elsewhere [36]. This subcohort was chosen for analysis, according to the fiber supplementation included in the study, similar to our MEATMARK cohort.

### Data analysis and statistics

Data were analyzed using R (v4.4.3) and GraphPad Prism (v10.1.2). Results are presented as mean ± SD (unless stated otherwise), and *p*-values < 0.05 were considered statistically significant (*). Normality was assessed using the Shapiro–Wilk test and Q-Q plots. Depending on the data distribution, either a paired t-test, Wilcoxon matched-pairs signed-rank test, or Mann-Whitney test was used for comparisons between two groups. For comparisons involving more than two groups, either an ordinary one-way ANOVA with Tukey’s multiple comparisons test or a Friedman test with Dunn’s multiple comparisons test was applied. The estimate of variation within each group is provided by the SD or standard error of the mean (SEM), as indicated under each figure or table. Additionally, variance was similar between groups, satisfying the assumption of homogeneity of variance.

A linear mixed-effects model (LMM) was fitted to the TMAO time-course data using the lmer() function (lme4 package, R). Plasma TMAO concentration (µM) served as the dependent variable. Fixed effects included Time (continuous, hours post-meal), its quadratic term (Time²), intervention group (fiber vs. placebo), meat intake frequency (regular vs. occasional), and their interactions. The model formula was: TMAO ~ (Time + Time²) * Group * Intake + (1 + Time | ID), with random intercepts and random slopes for time at the participant (ID) level. Parameters were estimated by restricted maximum likelihood (REML), and *p*-values were obtained using Satterthwaite’s approximation (lmerTest package). Model assumptions were assessed through residual diagnostics. Statistical significance was set at α = 0.05.

Only subjects with complete data sets were included. Therefore, no missing data had to be considered.

## Results

### Baseline characteristics of MEATMARK participants

Concerning inclusion and exclusion criteria, 24 volunteers were screened. Finally, 16 volunteers were enrolled. However, three participants dropped out during the study (Fig. 2). Thus, the final analysis dataset included 13 participants (6 females, 7 males) with a mean age of 28.6 ± 6.4 years and a mean BMI of 23.1 ± 2.4 kg/m^2^. A more comprehensive description of baseline characteristics is provided in Table S3**:** *Baseline characteristics* in the supplementary material. The dietary protocol analysis of the placebo and treatment intervention phases is shown in Table 1. Notably, total energy and macronutrient composition remained consistent across the two interventions. As expected, there was a statistically significant increase in total daily fiber intake during the treatment phase, which was 51.7 ± 7.56 g/day compared to 28.7 ± 5.45 g/day.

**Table 1:** Analysis of the dietary protocol.

	Placebo	Treatment	*p*-value
Energy intake [kcal/d]	2530 ± 724	2500 ± 649	ns
Carbohydrates [% total energy intake]	50.1 ± 5.11	48.1 ± 4.21	ns
Protein [% total energy intake]	11.8 ± 1.59	11.6 ± 1.76	ns
Fat [% total energy intake]	32.8 ± 4.11	32.4 ± 2.53	ns
Fiber total, [g/d]	28.7 ± 5.45	51.7 ± 7.56	0.01

Data is presented as mean ± SD. *P*-value < 0.05 was regarded as statistically significant; ns, not significant. According to normality distribution, either the paired *t*-test or the Wilcoxon-signed ranked test was applied to assess differences between placebo and treatment.

### TMAO as a beef intake biomarker

**Fig. 3:**
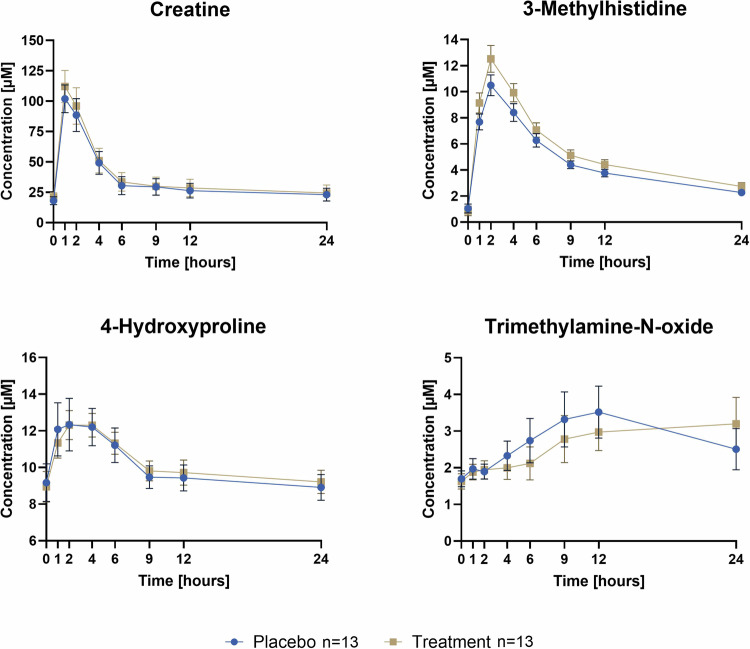
Plasma concentrations of meat intake biomarkers after beef consumption on the test day after 14 days of either fiber treatment or placebo intervention. Data is shown as mean ± SEM.

Figure 3 shows plasma concentrations for creatine, 3-methylhistidine, 4-hydroxyproline and TMAO in the MEATMARK cohort after the beef intake following the 14 days fiber or placebo interventions. Creatine, 3-methylhistidine and 4-hydroxyproline are validated biomarkers of meat intake [1, 21]. These four analytes were indicative of beef consumption, with plasma concentrations increasing after meat consumption and returning to baseline values after 24 h, except TMAO, which appears to remain in the blood for more than 24 h. Our results are in line with literature, as studies have shown that TMAO has a relatively long half-life in humans with approximately 6 to 12 h. TMAO levels can rise within a few hours post beef consumption and may remain elevated for up to 24 h [12, 31]. Pure descriptive analysis of the total group revealed no significant differences between the dietary fiber treatment and the placebo when comparing the maximum values and the AUC (see corresponding Table S4**:***AUC and maximum values of beef intake biomarkers* in the supplementary material). Furthermore, the fold change from baseline (0 h) to the individual maximum TMAO concentrations showed no significant difference between interventions (mean fold change difference [95% CI]: *−*0.26 [*−*1.4, 0.80], *p*-value = 0.26). Data are shown in Fig. S1 in the supplementary material.

### Occasional versus regular meat intake showed a different plasma TMAO response after two weeks of high-fiber intervention

**Fig. 4:**
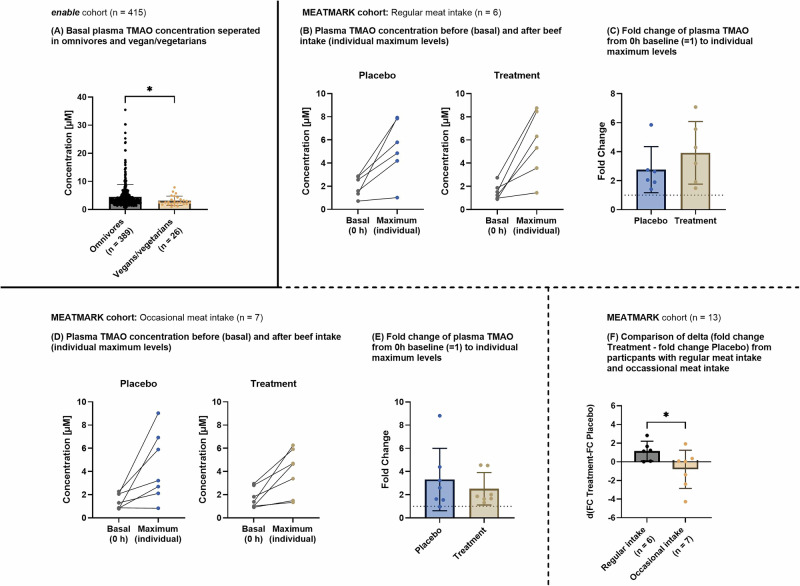
Plasma TMAO dynamics in diet groups and meat intake response. **A** Basal plasma TMAO concentrations in the enable cohort separated in omnivores and vegans/vegetarians. Data are presented as individual values (dots) and as mean ± SD (bar plot). According to normality distribution, the Mann-Whitney test was applied to assess differences between omnivores and vegans/vegetarians. **B** Plasma TMAO concentration before (basal) and after beef intake in the MEATMARK cohort from participants with regular meat intake. The respective individual maximum level within 24 h is given. Data is presented as individual values (dots). **C** Fold change of plasma TMAO from basal to individual maximum levels from participants with regular meat intake in the MEATMARK cohort. Data is presented as mean ± SD (bar plot). According to normality distribution, the Wilcoxon matched-pairs signed-rank test was applied to assess differences between placebo and treatment. **D** Plasma TMAO concentrations before (basal) and after beef intake in the MEATMARK cohort from participants with occasional meat intake. The respective individual maximum level within 24 h is shown. Data is presented as individual values (dots). **E** Fold change of plasma TMAO from basal to individual maximum levels from participants with occasional meat intake in the MEATMARK cohort. Data is presented as mean ± SD (bar plot). According to normality distribution, the Wilcoxon matched-pairs signed rank test was applied to assess differences between placebo and treatment. **F** Delta between FC placebo and treatment from participants with regular and occasional meat intake. Data is presented as mean ± SD (bar plot). According to normality distribution, the *t*-test was applied to assess differences between regular and occasional meat intake. For all panels, a *p*-value < 0.05 was regarded as statistically significant.

Given the known variations in TMAO metabolism between individuals adhering to vegetarian or omnivorous diets [12], we first aimed to explore these differences within the *enable* cohort in more detail. As shown in Fig. 4A, this study group revealed significant differences in basal plasma TMAO levels between vegetarians/vegans and omnivores (Hodges-Lehmann estimated median difference [95% CI]: *−*0.56 [*−*1.14 to 0.02], *p*-value = 0.028).

Although we were not able to confirm this effect in the MEATMARK cohort due to the small sample size (data not shown), we further stratified this group according to meat-eating frequency. Volunteers reporting meat consumption more than three times per week were classified as “regular” meat eaters, while those consuming meat three times or less were categorized as “occasional” meat eaters. Basal (i.e., before the beef test meal) and individual maximum plasma TMAO values (i.e., after beef consumption) for both groups were compared after treatment and placebo interventions (Fig. 4B/D). Interestingly, while maximum TMAO values appeared comparable between regular and occasional meat eaters after the placebo phase, a notable discrepancy emerged after the fiber intervention. Specifically, the increase in maximum TMAO values after beef consumption was less pronounced in the occasional meat-eater group compared to the regular meat-eater group. This effect was further elucidated by examining the FC of TMAO values (from basal to maximum value) within each intervention group (Fig. 4C/E). Notably, in the occasional meat eater group, the FC of TMAO decreased after the treatment intervention, in contrast to the observed increase in the regular meat eater group.

Furthermore, a comparative analysis of the delta effect of the FC between placebo and treatment revealed a significant difference between the regular and occasional meat eater groups (Fig. 4F). The occasional meat eater group exhibited a significantly greater reduction in TMAO levels after the fiber intervention compared to the regular meat-eating participants (mean difference [95% CI]: –1.96 [–4.00, 0.083], *p*-value = 0.029).

These findings underscore the differential response in TMAO levels to dietary fibers among individuals with varying meat consumption patterns, suggesting a potential approach for targeted dietary strategies to modulate TMAO metabolism.

To further support this finding, we performed a linear mixed-effects model (LMM) analysis incorporating a quadratic time structure, meat intake frequency, and intervention group as predictors. The LMM confirmed the observed trend of a greater reduction in TMAO response in occasional meat eaters after the fiber intervention. Model results and interpretation are presented in the Supplementary Material (Fig. S2).

### Dietary fiber intervention alters abundance of the TMA-producing microbial Enzyme *cutC*

**Fig. 5:**
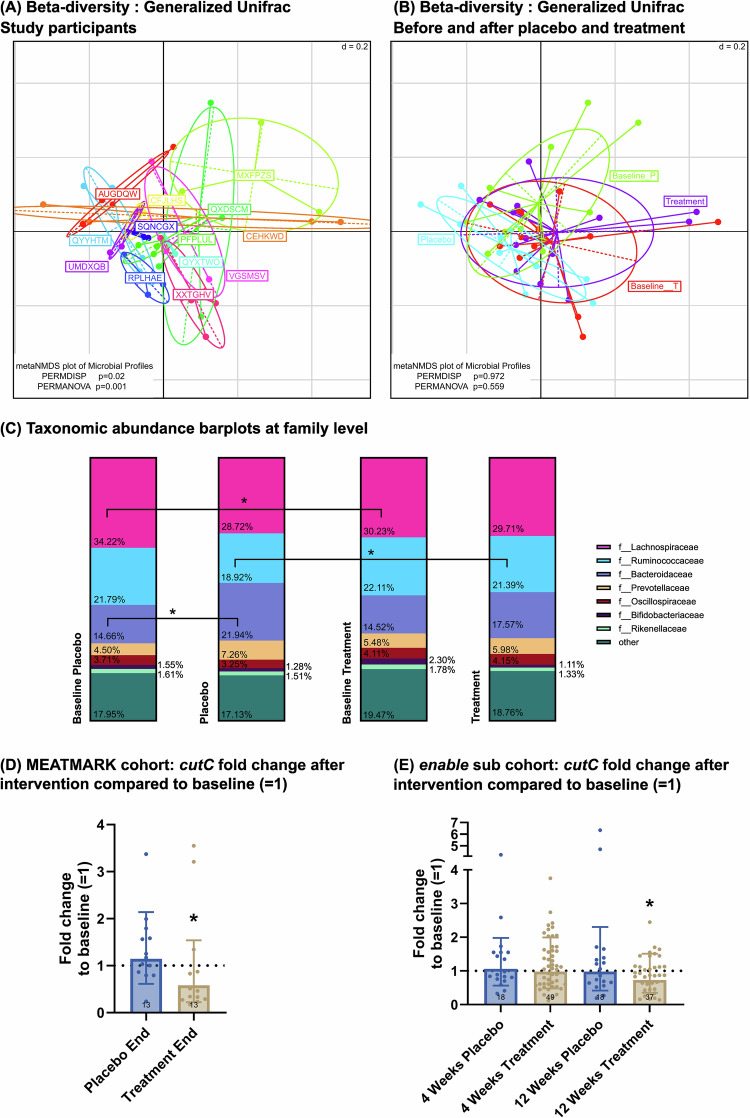
Fecal microbiota analysis by 16S rRNA gene amplicon analysis. **A** Beta diversity of individuals (every color represents one individual volunteer) and **B** at baseline and after two weeks of intervention with either placebo or treatment; PERMDISP and PERMANOVA were applied as statistical test. **C** Taxonomic bar plots on family level with relative abundance at baseline and after two weeks of placebo and treatment intervention. Data are presented as mean. According to normality distribution, either a one-way ANOVA with Tukey’s multiple comparisons test or the Friedman test with Dunn’s multiple comparisons test was applied. **D**, **E** Fold change of microbial cutC after placebo and treatment intervention compared to baseline fold change ( =1). Data is presented as mean ± SD (bar plot). According to normality distribution, either the one tailed paired *t*-test or one-tailed Wilcoxon matched-pairs signed rank test was applied to assess differences between fold change after intervention and baseline. The enable subgroup refers to the participants from the “Freising Fiber Acceptance Study”. For all panels, a *p*-value < 0.05 was regarded as statistically significant.

The analysis of the microbiome unveiled significant inter-individual differences in beta diversity (PERMANOVA *p*-value_gUniFrac_ < 0.001), indicating that microbial compositions among individuals differed noticeably (Fig. 5A). With regard to our primary research question of whether a fiber intervention affects the microbiome, we analyzed and compared the samples from the four sampling times (before and after placebo and treatment intervention). The average individual richness at baseline placebo was 55 ± 17 operational taxonomic units at species level (SOTUs), 60 ± 12 SOTUs at baseline treatment, 55 ± 14 SOTUs after placebo and 55 ± 17 SOTUs after treatment. The Shannon effective number of species was 30 ± 9 at baseline placebo, 34 ± 9 at baseline treatment, 30 ± 10 after placebo, and 31 ± 10 after treatment. Both, richness and Simpson effective numbers did not differ significantly between sampling times. Beta-diversity between sampling times also showed no significant difference, respectively no significant clusters were discernible based on the sampling times (Fig. 5B). The relative abundances of dominant gut microbiome phyla also remained remarkably consistent across all sampling times. Figure 5C illustrates the relative abundance of gut microbiota at family level at baseline and after placebo and treatment intervention, with *Lachnospiraceae*, *Ruminococcaceae*, *Bacteroidaceae*, *Prevotellaceae*, *Oscillospiraceae*, *Bifidobacteriaceae* and *Rikenellaceae* emerging as the predominant families. Between the two baseline samples, *Lachnospiraceae* was significant higher in the placebo baseline (*p*-value_*Lachnospiraceae*_ = 0.046). *Ruminococcaceae* was significant higher after treatment intervention compared to placebo intervention (*p*-value_*Ruminococcaceae*_ = 0.017) and *Bacteroidaceae* significantly increased compared to respective baseline after placebo intervention (*p*-value_*Bacteroidaceae*_ = 0.037).

Further analyses at the enzymatic level showed a change in the gene abundance of the enzyme *cutC* after the treatment intervention. Specifically, there was a significant decrease in *cutC* gene abundance after two weeks´ treatment compared to the respective baseline in MEATMARK (mean difference [95% CI]: 0.87 [–0.07, 1.81], *p*-value = 0.034; Fig. 5D). In the *enable* sub-cohort “Freising Fiber Acceptance Study” [36], a fiber intervention was conducted for four and twelve weeks, including a placebo. A significant decrease in *cutC* gene abundance after twelve weeks of dietary fiber intervention (mean difference [95% CI]: 0.23 [–0.16, 0.71], *p*-value = 0.016) was found (Fig. 5E**)**, but no change was observed after placebo.

Based on these findings, we performed a correlation analysis and found two OTUs, which may be related to an increase in *cutC* gene abundance. SOTU582 belongs to family *Lachnospiraceae* and SOTU411 belongs to family *Oscillospiraceae*, both indicate a higher *cutC* gene abundance (SOTU582: Pearson´s R: 0.868, *p*-value < 0.0001; SOTU411: Pearson´s R: 0.743, *p*-value < 0.0001). Figure S3 in the supplementary material depicts the correlation plots.

### Investigation of Three *FMO3* SNPs on Plasma TMAO Levels

When exploring the influence of various SNPs (rs909530, rs909531, rs2266780), no conclusive evidence was found for an association between these three specific genotypes and TMAO plasma levels. This analysis encompassed both the MEATMARK and enable [45] cohorts, with detailed results provided in Table 2.

**Table 2:** Basal and maximum TMAO plasma levels according to the *FMO3* SNP variation are shown.

*FMO3* SNP variation	*rs909530*			*p*-value
	*Wild type (CC)*	*Heterozygous (CT)*	*Mutation (TT)*	
*MEATMARK*, *n*	6	6	1	
Mean: basal TMAO levels [µM]	1.77 ± 0.57	1.47 ± 0.72	2.19	ns
Placebo: maximum TMAO levels [µM]	4.97 ± 2.61	4.11 ± 2.89	7.84	ns
Treatment: maximum TMAO levels [µM]	4.86 ± 1.20	4.00 ± 3.11	8.45	ns
*enable*, *n*	155	87	112	
Basal TMAO levels [µM]	3.25 ± 2.39	4.02 ± 4.46	3.92 ± 2.88	ns
	***rs909531***			
	***Wild type (TT)***	***Heterozygous (TC)***	***Mutation (CC)***	
*MEATMARK*, *n*	8	3	0	
Mean: basal TMAO levels [µM]	1.54 ± 0.62	1.42 ± 0.27	-	ns
Placebo: maximum TMAO levels [µM]	4.33 ± 3.20	5.68 ± 2.37	-	ns
Treatment: maximum TMAO levels [µM]	4.60 ± 2.45	4.14 ± 2.45	-	ns
*enable*, *n*	168	79	7	
Basal TMAO levels [µM]	3.24 ± 2.31	4.13 ± 4.67	4.29 ± 3.31	ns
	***rs2266780***			
	***Wildtype (AA)***	***Heterozygous (AG)***	***Mutation (GG)***	
*MEATMARK*, *n*	10	3	0	
Mean: basal TMAO levels [µM]	1.74 ± 0.70	1.42 ± 0.27	-	ns
Placebo: maximum TMAO levels [µM]	4.53 ± 2.87	5.68 ± 2.37	-	ns
Treatment: maximum TMAO levels [µM]	4.91 ± 2.57	4.14 ± 2.45	-	ns
*enable*, *n*	169	78	7	
Basal TMAO levels [µM]	3.22 ± 2.30	4.16 ± 4.70	4.50 ± 3.52	ns

Data is presented as mean ± SD. According to normality distribution, the Wilcoxon-signed ranked test was applied to assess differences in TMAO levels between meat intake frequency and sex. *P*-value < 0.05 was regarded as statistically significant. MEATMARK: in all three SNPs the wild type and heterozygous were statistically compared with a Wilcoxon-sign rank test. *enable*: For the statistical analysis, a Kruskal-Wallis multiple comparison test was used. Since none of the variations showed a significant difference, the *p*-values re*p*resents multiple comparison analysis. *ns* not significant.

## Discussion

The precise origin and regulation of TMAO in response to dietary intake remains incompletely understood. With data from the MEATMARK study, we contribute to understanding the effects of endogenous and exogenous factors on TMAO formation.

Our findings confirm TMAO as a biomarker of beef consumption, and we investigated whether a dietary fiber intervention could modify postprandial TMAO levels following beef intake. While no effect was observed across the entire study cohort, stratification into “occasional” and “regular” meat-eaters revealed a lower postprandial TMAO increase in occasional meat-eaters who consumed dietary fiber.

Additionally, in a separate analysis, we observed significantly lower fasting-state plasma TMAO levels in vegetarians compared to omnivores in the *enable* cohort [45]. This aligns with previous studies, reinforcing that habitual dietary patterns influence TMAO metabolism [12]. It is important to note that, unlike the MEATMARK cohort, participants in the *enable* cohort were not subject to dietary restrictions before blood sampling. As a result, TMAO precursors may have originated from multiple dietary sources, including fish, eggs, beetroot, and mushrooms, rather than exclusively from meat intake [1, 2, 13].

### Dietary patterns and TMAO metabolism

TMAO remains a subject of debate as a cardiovascular risk factor [9, 10, 13], and dietary patterns may play a key role in modulating its levels. A growing body of evidence underscores the significant impact of diet in cardiovascular morbidity and mortality [46, 47]. Key dietary risk factors include high intakes of saturated fat and sodium, as well as low fiber consumption. Additionally, excessive meat intake, particularly processed meat, has been linked to increased cardiovascular risk [48].

Our findings suggest that habitual meat consumption strongly influences TMAO formation. We found that dietary fiber intake mitigated postprandial TMAO formation in occasional meat-eaters but not in regular meat-eaters. This aligns with prior findings by Koeth et al., who reported that individuals with lower meat consumption exhibited lower postprandial TMAO production [12]. They hypothesized that vegans and vegetarians have a reduced capacity for TMAO formation due to microbial adaptation to their habitual diet.

Our findings support this hypothesis and suggest that habitual dietary patterns condition the gut microbiome for TMAO production. This underscores the potential cardiovascular benefits of a diet rich in fiber and low in meat. Thus, one promising approach to reducing CVD risk through decreased TMAO levels could be a diet low in meat and high in fiber.

### Microbiome adaptation and TMAO formation

Our microbiome analysis further highlights the potential modulatory effects of dietary fiber on TMAO metabolism. Dietary fibers have been widely recognized for their health-promoting effects, including contributions to immune homeostasis, metabolic regulation, glucose homeostasis, intestinal barrier integrity, and appetite control [26]. These effects are largely mediated by prebiotic functions [49]. Given the well-documented positive effects of dietary fiber on gut microbiota, we investigated its functional impact on microbial TMA formation.

While our study revealed some microbiome changes at the family level following both the placebo and dietary fiber interventions, the specific effects of these changes remain uncertain. Notably, even minor food-processing variations have been shown to influence microbiome composition in animal models [50], suggesting that the gut microbiota is highly adaptable [51]. Bacterial phyla such as Bacillota, Actinomycetota, and Pseudomonadota have been identified as key contributors to TMA formation, though not every bacterium within these groups possesses TMA-producing capacity [15, 25, 26]. Therefore, we focused on *cutC* gene abundance, which encodes the choline TMA-lyase enzyme responsible for TMA production [12].

Remarkably, in the MEATMARK cohort, global *cutC* gene abundance significantly decreased after two weeks of dietary fiber intervention but remained unchanged after placebo intervention. A similar pattern was observed in the *enable* cohort after a twelve-week dietary fiber intervention [36].

Furthermore, correlation analysis in the MEATMARK cohort identified two significant species-level operational taxonomic units (SOTUs) potentially linked to *cutC* abundance; one from the Lachnospiraceae family and another from the Oscillospiraceae family. This suggests that dietary fiber interventions can modulate microbial pathways relevant to TMAO metabolism, reinforcing the beneficial role of fiber-rich diets. Future research should investigate these SOTUs in greater depth and to further explore how microbiome profiles interact with meat consumption.

### Genetic variability (*FMO3*) in TMAO metabolism

Our study also aimed to investigate the role of *FMO3* genetic variants in TMAO metabolism; however, no clear genotype associations were detectable in our cohorts. The limited sample size in the MEATMARK study and the lack of dietary standardization in the *enable* cohort may have contributed to this result. Given the interesting findings from mechanistic studies in mouse models, where *FMO3* suppression reduced TMAO levels and atherosclerosis [32], further research in larger and more standardized human studies is needed to better understand the genetic regulation of TMAO metabolism and its role in CVD risk.

### Study strengths and limitations

A key strength of this study is the replication of findings in an independent cohort (*enable* cohort), supporting the robustness of our results. However, certain limitations must be considered. One important limitation of our study is the relatively small sample size, which limits the statistical power to detect potentially meaningful differences between intervention groups. As a result, some observed trends did not reach formal statistical significance. Additionally, the post-hoc stratification into occasional and regular meat eaters, which was not part of the original study design, may have introduced potential bias and should be interpreted with caution. However, the consistent direction of effects across two analytical approaches, delta-FC-based and time-resolved mixed-effects modeling, as well as across different study cohorts, suggests these trends may still reflect biologically relevant responses. Future studies with larger sample sizes will be needed to validate these findings and to enable more detailed modeling of potential period and carry-over effects.

Additionally, the dose and duration of fiber intervention may influence results. Although participants might have received a higher dose of insoluble fiber; however, we prioritized maintaining a physiologically relevant intake. On average, participants consumed 51 g of fiber per day (from both diet and supplementation), a dose that has been shown in previous studies to significantly impact gut health and induce measurable microbiome changes [52–55]. For instance, O’Keefe et al. demonstrated that consuming approximately 50 g of fiber per day led to substantial improvements in intestinal mucosal biomarkers within just two weeks [52]. Nevertheless, variations in fiber type, dosage, and intervention duration should be considered in future larger-scale studies to refine dietary recommendations.

## Conclusion

The MEATMARK study demonstrated that supplementing a regular diet with 27 g of dietary fiber modulates plasma TMAO formation, but only in individuals with a low habitual meat intake (<3 portions per week). Furthermore, dietary fiber intake appears to influence microbiome composition, particularly *cutC* gene abundance. These findings suggest that reducing meat intake while increasing fiber consumption may mitigate TMAO production, potentially reducing cardiovascular risk. However, additional studies are needed to further investigate TMAO’s role in CVD risk and the mechanistic pathways through which fiber modulates TMAO metabolism. Future research should focus on larger cohorts, longer intervention periods, and controlled dietary intake to better understand the long-term impact of dietary modifications on TMAO formation and cardiovascular health.

## Supplementary information

Supplemental Material_2

## Data Availability

Data described in the manuscript will be made available upon request.
