# Supplementary material for: Effect of dietary fiber on trimethylamine-N-oxide production after beef consumption and on gut microbiota: MEATMARK – a randomized cross-over study
Source: Eur J Clin Nutr. 2025 Jun 19;79(10):980–90. doi: 10.1038/s41430-025-01636-8 (PMC12537495; doi:10.1038/s41430-025-01636-8)
Supplement: Supplementary file 1 — Supplemental Material_2 [file 41430_2025_1636_MOESM1_ESM.docx]

**Supplemental material**

**Table S1: Composition of study fiber supplements (%)**

|  | **Dietary fiber-enriched drink (Treatment)** | | | **Placebo drink** | | |
| --- | --- | --- | --- | --- | --- | --- |
| **Component** | **Cherry (%)** | **Multi-Fruit (%)** | **Blackcurrant (%)** | **Cherry (%)** | **Multi-Fruit (%)** | **Blackcurrant (%)** |
| VITACEL wheat plant fiber | 45.00 | 45.00 | 45.00 | 3.75 | 3.75 | 3.75 |
| VITACEL psyllium | 3.75 | 3.75 | 3.75 | 3.75 | 3.75 | 3.75 |
| Guar | 3.75 | 3.75 | 3.75 | 3.75 | 3.75 | 3.75 |
| Isomaltulose | 42.00 | 43.65 | 43.875 | 50.75 | 52.40 | 53.25 |
| Sucralose | 0.125 | 0.10 | 0.125 | 0.125 | 0.10 | 0.125 |
| Citric acid | 4.125 | 2.50 | 2.50 | 4.125 | 2.50 | 1.875 |
| Flavor cherry | 0.875 | 0.00 | 0.00 | 0.875 | 0.00 | 0.00 |
| Flavor multi-fruit | 0.00 | 0.875 | 0.00 | 0.00 | 0.875 | 0.00 |
| Flavor blackcurrant | 0.00 | 0.00 | 0.625 | 0.00 | 0.00 | 0.625 |
| Ponceau 4R E124 | 0.375 | 0.00 | 0.375 | 0.375 | 0.00 | 0.375 |
| Beta-carotene | 0.00 | 0.375 | 0.00 | 0.00 | 0.375 | 0.00 |
| Maltodextrine | 0.00 | 0.00 | 0.00 | 25.00 | 25.00 | 25.00 |
| Vegetable potato flakes | 0.00 | 0.00 | 0.00 | 7.50 | 7.50 | 7.50 |

**Table S2: MRM transitions for quantification of amino acids and acylcarnitines**

| **Q1 (m/z)** | **Q3 (m/z)** | **RT (min)** | **Name** | **DP declustering potential (V)** | **EP entrance potential (V)** | **CE collision energy (V)** | **CXP cell exit potential (V)** |
| --- | --- | --- | --- | --- | --- | --- | --- |
| 69.03 | 49.2 | 4.14 | IS trimethylamine-d9 TMA-d9 | 101 | 10 | 31 | 8 |
| 85.031 | 68.2 | 4.9 | IS trimethylamine oxide d9 tmao-d9 | 106 | 10 | 17 | 10 |
| 104.847 | 60.1 | 4.25 | Choline 104 | 31 | 10 | 25 | 6 |
| 104.847 | 45 | 4.25 | Choline 104 | 31 | 10 | 47 | 4 |
| 113.009 | 69.2 | 4.25 | IS choline-d9 | 31 | 10 | 25 | 6 |
| 113.918 | 44.2 | 2.91 | Creatinine | 71 | 10 | 25 | 6 |
| 113.918 | 43.2 | 2.91 | Creatinine | 71 | 10 | 53 | 6 |
| 117.912 | 72.1 | 10.44 | Guanidinoacetic acid | 41 | 10 | 19 | 10 |
| 117.912 | 43.2 | 10.44 | Guanidinoacetic acid | 41 | 10 | 47 | 6 |
| 137.944 | 65.1 | 1.01 | AA | 56 | 10 | 57 | 6 |
| 137.944 | 92 | 1.01 | AA | 56 | 10 | 35 | 54 |
| 153.95 | 136.1 | 1.27 | Hydroxy anthranilic acid | 51 | 10 | 17 | 6 |
| 153.95 | 80.1 | 1.27 | Hydroxy anthranilic acid | 51 | 10 | 35 | 12 |
| 169.985 | 124 | 11.54 | Tau-Methyl histidine | 56 | 10 | 19 | 16 |
| 173.002 | 127.1 | 11.54 | IS d3-tau-methyl-histidine | 66 | 10 | 21 | 4 |
| 175.971 | 129.9 | 1.05 | IAA | 106 | 10 | 27 | 16 |
| 175.971 | 103 | 1.04 | IAA | 106 | 10 | 45 | 12 |
| 180.953 | 164.1 | 5.22 | IS serotonin-d4 | 56 | 10 | 17 | 8 |
| 189.016 | 84.1 | 12.2 | Trimethyllysine | 76 | 10 | 29 | 12 |
| 189.016 | 56.1 | 12.2 | Trimethyllysine | 76 | 10 | 53 | 8 |
| 189.981 | 129.9 | 0.98 | IPA | 96 | 10 | 23 | 16 |
| 189.981 | 77.1 | 0.98 | IPA | 96 | 10 | 89 | 6 |
| 198.094 | 84.1 | 12.2 | IS trimetyllysine-d9 tml-d9 | 81 | 10 | 29 | 12 |
| 198.094 | 130.1 | 12.2 | IS trimetyllysine-d9 tml-d9 | 81 | 10 | 19 | 4 |
| 205.886 | 160.1 | 6.48 | Xanthurenic Acid | 131 | 10 | 25 | 12 |
| 205.886 | 132.1 | 6.48 | Xanthurenic Acid | 131 | 10 | 41 | 4 |
| 221.003 | 133.9 | 9.05 | 5-Hydroxytryptophan | 31 | 10 | 35 | 16 |
| 221.003 | 160 | 9.05 | 5-Hydroxytryptophan | 31 | 10 | 25 | 20 |
| 224.973 | 110 | 8.48 | Hydroxykynurenine | 56 | 10 | 25 | 12 |
| 224.973 | 162 | 8.48 | Hydroxykynurenine | 56 | 10 | 27 | 24 |
| 227.009 | 155.9 | 12.46 | Carnosine | 116 | 10 | 23 | 18 |
| 227.009 | 93 | 12.46 | Carnosine | 116 | 10 | 51 | 4 |
| 232.052 | 85 | 5.58 | Isobutyrylcarnitine | 36 | 10 | 51 | 10 |
| 232.052 | 173.1 | 5.58 | Isobutyrylcarnitine | 36 | 10 | 19 | 8 |
| 241.012 | 109.1 | 12.43 | Anserine | 56 | 10 | 31 | 16 |
| 241.012 | 95.1 | 12.43 | Anserine | 56 | 10 | 63 | 14 |
| 246.076 | 187.1 | 5.18 | 2-Methylbutyrycarnitine | 56 | 10 | 19 | 8 |
| 246.076 | 60.1 | 5.18 | 2-Methylbutyrycarnitine | 56 | 10 | 45 | 10 |
| 176.96 | 160 | 5.22 | Serotonin | 106 | 10 | 13 | 22 |
| 176.96 | 132.1 | 5.22 | Serotonin | 106 | 10 | 29 | 16 |
| 204.054 | 186 | 0.95 | Indole-3-butyric acid | 81 | 10 | 19 | 8 |
| 204.054 | 129.9 | 0.95 | Indole-3-butyric acid | 81 | 10 | 35 | 18 |
| 170.024 | 170.2 | 11.54 | Tau-methyl-l-histidine | 46 | 10 | 9 | 8 |
| 170.024 | 124.1 | 11.54 | Tau-methyl-l-histidine | 46 | 10 | 19 | 18 |
| 75.898 | 76 | 4.95 | TMAO_neu | 176 | 10 | 9 | 12 |
| 59.98 | 44 | 4.14 | Trimethylamine_TMA_neu | 71 | 10 | 27 | 4 |
| 59.98 | 45 | 4.14 | Trimethylamine_TMA_neu | 71 | 10 | 19 | 6 |
| 178.01 | 132.1 | 1.04 | IS d2 IAA | 96 | 10 | 25 | 4 |
| 173.029 | 127.1 | 11.54 | IS d3Tau-M-His | 71 | 10 | 21 | 4 |
| 78.144 | 32.1 | 10.65 | IS Glycin (2,2-D2)_quan | 1 | 10 | 21 | 14 |
| 90.164 | 30 | 9.44 | Sarcosine_qual | 46 | 10 | 59 | 14 |
| 90.164 | 44 | 9.44 | Sarcosine_quan | 46 | 10 | 15 | 20 |
| 90.173 | 30.1 | 10.26 | Beta-Alanin_qual | 36 | 10 | 25 | 14 |
| 90.173 | 72 | 10.26 | Beta-Alanin_quan | 36 | 10 | 11 | 32 |
| 92.981 | 45.9 | 10.07 | IS Alanin (13C3)_quan | 1 | 10 | 13 | 12 |
| 106 | 60 | 11.12 | Serine 1 Q | 41 | 10 | 17 | 4 |
| 106 | 88 | 11.12 | Serine 2 | 41 | 10 | 13 | 6 |
| 132.1 | 86 | 6.96 | Leucine 1 Q | 81 | 10 | 15 | 14 |
| 138.998 | 92 | 12.35 | IS L-Asparatic acid (13C4_15N) 2 | 36 | 10 | 15 | 14 |
| 147.013 | 84 | 11.07 | Glutamine 1 Q | 66 | 10 | 23 | 10 |
| 152.2 | 88 | 11.07 | IS Glutamine (13C5) 1 Q | 66 | 10 | 23 | 10 |
| 205.1 | 188.1 | 7.09 | Tryptophan Q 1 | 51 | 10 | 17 | 4 |
| 205.1 | 146 | 7.09 | Tryptophan 2 | 31 | 10 | 23 | 8 |
| 210.2 | 192.1 | 7.09 | IS Tryptophan (indole-d5) Q 1 | 51 | 10 | 17 | 4 |
| 161.999 | 84.9 | 9.41 | C0_qual | 76 | 10 | 25 | 10 |
| 161.999 | 103 | 9.41 | C0_quan | 76 | 10 | 21 | 12 |
| 165.025 | 103 | 9.41 | IS C0 (N-methyl-d3)_quan | 81 | 10 | 21 | 12 |
| 204.039 | 145 | 7.4 | C2_qual | 61 | 10 | 17 | 16 |
| 204.039 | 84.9 | 7.4 | C2_quan | 61 | 10 | 23 | 10 |
| 207.158 | 85 | 7.4 | IS C2 (N-methyl-d3)_quan | 56 | 10 | 23 | 10 |
| 218.052 | 159 | 6.32 | C3_qual | 71 | 10 | 17 | 18 |
| 218.052 | 85 | 6.32 | C3_quan | 71 | 10 | 23 | 10 |
| 221.123 | 84.9 | 6.32 | IS C3 (N-methyl-d3)_quan | 56 | 10 | 25 | 10 |
| 232.034 | 173 | 5.63 | C4_qual | 71 | 10 | 17 | 20 |
| 232.034 | 84.9 | 5.63 | C4_quan | 71 | 10 | 23 | 10 |
| 235.141 | 84.9 | 5.63 | IS C4 (N-methyl-d3)_quan | 61 | 10 | 25 | 12 |
| 244.081 | 185.1 | 5.47 | C5-1 Tigloyl_qual | 61 | 10 | 17 | 20 |
| 244.081 | 85 | 5.47 | C5-1 Tigloyl_quan | 61 | 10 | 25 | 12 |
| 246.161 | 187 | 5.17 | iC5_qual | 61 | 10 | 17 | 22 |
| 246.161 | 84.9 | 5.17 | iC5_quan | 61 | 10 | 25 | 10 |
| 248.122 | 144.1 | 8.5 | C3DC_qual | 81 | 10 | 27 | 16 |
| 248.122 | 84.9 | 8.5 | C3DC_quan | 81 | 10 | 27 | 10 |
| 248.145 | 103 | 8.47 | C4-OH_qual | 66 | 10 | 25 | 12 |
| 248.145 | 85 | 8.47 | C4-OH_quan | 66 | 10 | 25 | 10 |
| 260.08 | 201 | 4.8 | C6_qual | 91 | 10 | 17 | 24 |
| 260.08 | 84.9 | 4.8 | C6_quan | 91 | 10 | 25 | 10 |
| 262.08 | 84.6 | 7.89 | C4DC_neu_qual | 66 | 10 | 49 | 10 |
| 262.08 | 84.9 | 7.89 | C4DC_neu_quan | 66 | 10 | 27 | 10 |
| 262.102 | 145 | 7.89 | 3-OH-iC5_qual | 66 | 10 | 21 | 16 |
| 262.102 | 84.9 | 7.89 | 3-OH-iC5_quan | 66 | 10 | 27 | 10 |
| 262.142 | 60 | 7.89 | C4brDC_qual2 | 81 | 10 | 21 | 8 |
| 262.142 | 84.9 | 7.89 | C4brDC_quan | 81 | 10 | 27 | 10 |
| 263.15 | 84.9 | 4.8 | IS C6 (N-methyl-d3)_quan | 71 | 10 | 25 | 10 |
| 265.24 | 85 | 7.89 | IS C4DC (N-methyl-d3)_quan | 56 | 10 | 31 | 42 |
| 276.03 | 198.9 | 9.86 | C5DC_qual | 81 | 10 | 23 | 24 |
| 276.03 | 84.9 | 9.86 | C5DC_quan | 81 | 10 | 27 | 10 |
| 288.13 | 229 | 4.45 | C8_qual | 81 | 10 | 19 | 24 |
| 288.13 | 85 | 4.45 | C8_quan | 81 | 10 | 27 | 10 |
| 291.201 | 84.9 | 4.45 | IS C8 (N-methyl-d3)_quan | 71 | 10 | 27 | 10 |
| 316.11 | 257 | 4.22 | C10_qual | 86 | 10 | 19 | 16 |
| 316.11 | 85 | 4.22 | C10_quan | 86 | 10 | 27 | 10 |
| 344.133 | 285.1 | 4.03 | C12_qual | 116 | 10 | 21 | 34 |
| 344.133 | 84.9 | 4.03 | C12_quan | 116 | 10 | 29 | 10 |
| 347.323 | 85 | 4.03 | IS C12 (N-methyl-d3)_quan | 56 | 10 | 27 | 12 |
| 372.377 | 313.2 | 3.86 | C14_qual | 116 | 10 | 21 | 38 |
| 372.377 | 84.9 | 3.86 | C14_quan | 116 | 10 | 29 | 10 |
| 375.333 | 84.9 | 3.86 | IS C14 (N-methyl-d3)_quan | 136 | 10 | 27 | 10 |
| 400.236 | 341.1 | 3.71 | C16_qual | 1 | 10 | 19 | 38 |
| 400.236 | 85 | 3.71 | C16_quan | 1 | 10 | 29 | 10 |
| 424.387 | 84.9 | 3.64 | C18-2-9Z-12Z_quan | 116 | 10 | 29 | 10 |
| 426.406 | 84.6 | 3.53 | C18-1-9Z_qual | 106 | 10 | 87 | 10 |
| 426.406 | 84.9 | 3.53 | C18-1-9Z_quan | 106 | 10 | 31 | 10 |
| 428.279 | 369.2 | 3.53 | C18_qual | 106 | 10 | 23 | 4 |
| 428.279 | 85 | 3.53 | C18_quan | 106 | 10 | 27 | 4 |
| 431.423 | 84.9 | 3.53 | IS C18 (N-methyl-d3)_quan | 116 | 10 | 29 | 10 |
| 444.366 | 145 | 4.55 | 3R-3-OH-C18_qual | 131 | 10 | 29 | 16 |
| 444.366 | 85 | 4.55 | 3R-3-OH-C18_quan | 131 | 10 | 31 | 10 |

**Table S3: Baseline characteristics**

Data is presented as mean ± SD.

|  | **Female** | **Male** | **Total** |
| --- | --- | --- | --- |
| n | 6 | 7 | 13 |
| Age (yrs) | 23.8 ± 4.5 | 32.7 ± 4.7 | 28.6 ± 6.4 |
| Weight (kg) | 61.9 ± 8.1 | 77.2 ± 12.9 | 70.2 ± 12.6 |
| Height (cm) | 166.0 ± 3.8 | 180.0 ± 8.2 | 174.0 ± 9.7 |
| BMI (kg/m^2^) | 22.5 ± 1.9 | 23.6 ± 2.8 | 23.1 ± 2.4 |

**Table S4: AUC and maximum values of beef intake biomarkers**

Data is presented as mean ± SD. According to normality distribution, either the paired t‐test or the Wilcoxon matched-pairs signed rank test was applied to assess differences between AUC treatment and AUC placebo and maximum value treatment and maximum value placebo. The maximum TMAO value is the mean from all individual maximum levels within 24 hours after beef consumption. *P*‐value < 0.05 was regarded as statistically significant.

| **Abbr.** | **AUC Treatment [µM*h]** | **AUC Placebo [µM*h]** | ***p*-value** |
| --- | --- | --- | --- |
| TMAO | 64.7 ± 31.2 | 68.6 ± 33.8 | 0.64 |
| Creatine | 903.1 ± 669.5 | 847.3 ± 587.4 | 0.45 |
| Methylhistidine | 130.7 ± 37.2 | 110.9 ± 28.5 | 0.17 |
| Hydroxyproline | 244.8 ± 53.4 | 240.5 ± 66.5 | 0.59 |
| **Abbr.** | **Maximum value Treatment [µM]** | **Maximum value Placebo [µM]** | ***p*-value** |
| TMAO | 4.7 ± 2.5 | 4.8 ± 2.7 | 0.90 |
| Creatine | 117.6 ± 49.5 | 107.6 ± 42.5 | 0.23 |
| Methylhistidine | 12.6 ± 3.7 | 10.6 ± 2.8 | 0.13 |
| Hydroxyproline | 13.09 ± 2.7 | 13.5 ± 4.9 | 0.79 |

**Figure S1**


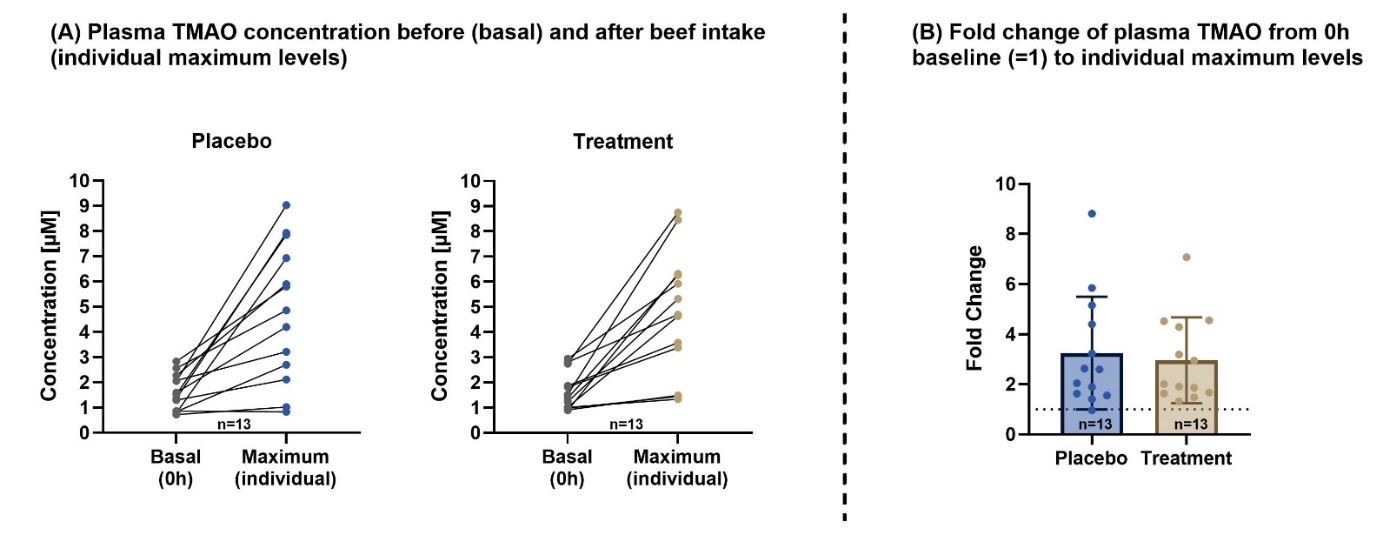


**Figure S1 A** Plasma TMAO concentration before (basal) and after beef intake in the whole MEATMARK cohort (n = 13). The respective individual maximum level within 24 hours is given. **B** The fold change (FC) of plasma TMAO from basal to individual maximum According to normality distribution the paired t-test was applied to assess differences between the FC of TMAO from baseline to individual maximum level after placebo and treatment intervention. P‐values < 0.05 were regarded as statistically significant.

**Figure S2**

**
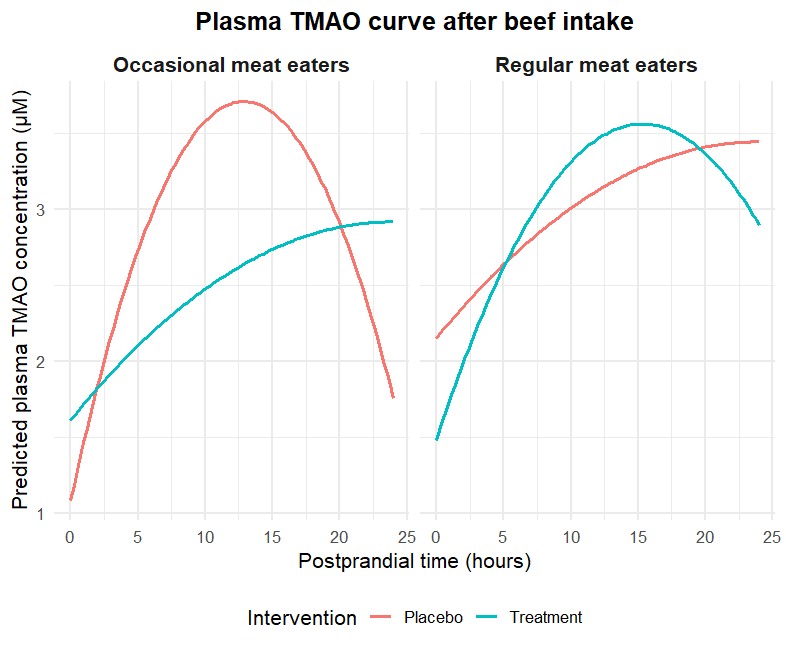
**

**Figure S2** Plasma TMAO curves after beef intake under placebo and fiber intervention, modeled using a quadratic linear mixed-effects model (LMM). Predicted plasma TMAO concentrations over time are shown separately for occasional meat eaters (n= 7) and regular meat eaters (n=6). Solid lines represent the model-fitted curve for the placebo (red) and fiber treatment (blue) interventions.

**Results and Interpretation of LMM:**

A linear mixed-effects model incorporating both linear and quadratic time effects was fitted to characterize postprandial plasma TMAO concentrations. The model revealed a significant positive linear time effect (regression coefficient (β) = 0.41, SE = 0.085, p < 0.001) and a significant negative quadratic time effect (β = –0.016, SE = 0.0033, p < 0.001), confirming a curvilinear trajectory with an initial rise and subsequent fall in TMAO levels after beef consumption.

There were no significant main effects of the intervention (fiber treatment vs. placebo; β = 0.53, SE = 0.46, p = 0.25) or meat intake frequency (regular vs. occasional; β = 1.06, SE = 0.75, p = 0.17) when averaged across time.

However, several significant interaction effects were detected:

- Time × Intervention_Group interaction (β = –0.30, SE = 0.11, p = 0.0097),
- Time² × Intervention_Group interaction (β = 0.014, SE = 0.0046, p = 0.0037),
- Time × Meat_Intake interaction (β = –0.30, SE = 0.12, p = 0.018),
- Time² × Meat_Intake interaction (β = 0.014, SE = 0.0048, p = 0.0054),
- and, the three-way interaction Time × Group × Intake (β = 0.46, SE = 0.17, p = 0.0068) and Time² × Intervention_Group × Meat_Intake (β = –0.020, SE = 0.0068, p = 0.0035).

These interactions indicate that the trajectory of TMAO concentrations over time differed depending on both the intervention and habitual meat intake. Specifically, occasional meat eaters in the fiber intervention group exhibited a less pronounced TMAO increase and a faster decline compared to regular meat eaters, suggesting a beneficial modulation of TMAO responses by fiber predominantly in the occasional meat eater subgroup.

These results align with findings from our delta-based analysis (delta between FC placebo and treatment) reported in 3.3., supporting the biological relevance of the observed trend.

**Figure S3**

**
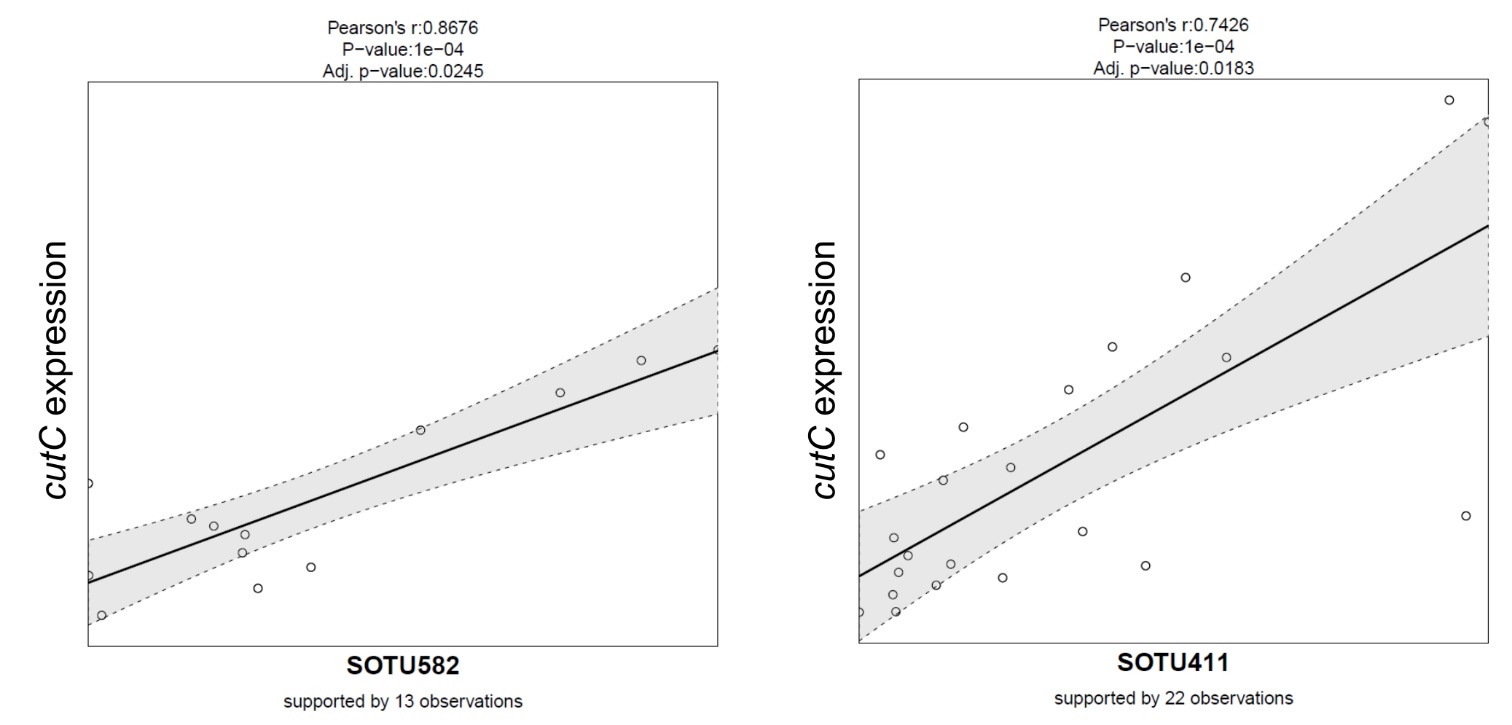
**

*cutC* gene abundance

*cutC* gene abundance

**Figure S2:** Correlation plots of SOTUs and *cutC* gene abundance. Pearson´s correlation coefficient was calculated.
